# Assessment of Food Safety and Practices in Nutrition Services: Case Study of Al-Ahsa Hospitals

**DOI:** 10.3390/healthcare13141723

**Published:** 2025-07-17

**Authors:** Randah Miqbil Alqurashi, Arwa Ibrahim Al-Humud

**Affiliations:** Department of Food Science and Nutrition, College of Agriculture and Food Sciences, King Faisal University, Al-Ahsa 31982, Saudi Arabia

**Keywords:** food safety, nutrition services, hygiene practices, cross-contamination, food storage, hospital staff training, Al-Ahsa, Saudi Arabia

## Abstract

**Background/Objectives:** This study assessed Knowledge and Practices related to Food Safety (KPFS) among nutrition services employees in hospitals across the Al-Ahsa Governorate, Kingdom of Saudi Arabia. The objective was to evaluate the staff’s understanding of key food safety principles, including foodborne illness prevention, food handling, personal hygiene, and food storage and preparation practices. **Methods**: A descriptive survey method was used, and data were collected using an electronic questionnaire, which was either self-administered by the participants or completed with the assistance of the researcher in cases involving employees who did not speak Arabic or English. This study included 302 staff members involved in the preparation, service, and supervision of food provided to hospital patients. **Results**: The results indicated a high level of knowledge among nutrition services employees regarding food safety principles, critical temperature control, cross-contamination prevention, and proper hygiene practices. The employees also demonstrated a strong commitment to personal hygiene behaviors, such as handwashing, wearing appropriate clothing, and avoiding unsafe practices. Additionally, a high degree of knowledge and understanding was found regarding food storage procedures and contamination prevention. The study also highlighted a very high level of awareness concerning the cleaning and sterilization of equipment, tools, and food storage surfaces, as well as maintaining a clean and healthy environment. These findings emphasize the importance of continuous training in enhancing food safety knowledge among nutrition services employees. **Conclusions**: It is recommended that all employees, regardless of education level, experience, or role, participate regularly in food safety training programs to sustain and improve food safety practices within hospital environments.

## 1. Introduction

Food safety is a critical public health concern that demands strict attention across all food service establishments, including hospitals. It refers to the proper handling, preparation, and storage of food to prevent contamination by harmful chemicals or pathogenic microorganisms. Ensuring food safety reduces the risk of foodborne illnesses, which are transmitted through contaminated food and pose serious health threats [1]. Nutrition and food safety are intrinsically linked; unsafe food perpetuates a vicious cycle of foodborne diseases, particularly affecting vulnerable groups such as infants, young children, the elderly, and immunocompromised individuals. According to 2017 estimates by the Centers for Disease Control and Prevention (CDC), approximately 50 million Americans suffer from foodborne illnesses annually, resulting in approximately 3000 deaths [2]. Similarly, the World Health Organization (WHO) reported that children under five years of age account for 40% of the global burden of foodborne diseases [3].

Despite increasing awareness of these risks, food safety remains marginalized in many healthcare systems [4]. Food hygiene and safety continue to be a pressing global challenge, especially in developing countries where poor hygiene practices, limited infrastructure, and weak regulatory systems exacerbate the problem [5,6]. The WHO has identified poor food-handling practices, inadequate sanitation, insufficient legislation, and financial constraints as major contributors to the prevalence of foodborne illnesses in these regions. Foodborne diseases also persist in developed nations, highlighting food safety as an issue of worldwide significance [7].

Over the past few decades, food poisoning has emerged as a growing public health threat globally. Defined as illnesses acquired through the consumption of contaminated food or water, food poisoning can result from various sources, including bacteria, viruses, toxic chemicals, radioactive substances, and other harmful agents. Symptoms may range from mild gastrointestinal discomfort to severe complications leading to disability or death. Many cases are linked to unsafe food practices such as improper storage, reheating, or cross-contamination. Research indicates that up to 50% of foodborne illnesses result from improper food storage, 45% from inappropriate storage practices, and 39% from cross-contamination [8,9,10]. Thus, improving food handlers’ knowledge, attitudes, and practices is key to reducing the incidence of foodborne illnesses in food service operations [1]. Several studies have highlighted lower rates of food safety problems in some countries, such as Jordan and Saudi Arabia, where reported issues range between 10% and 19.31% [11,12]. However, the global burden remains high, with the WHO estimating that one in ten people falls ill each year due to unsafe food. The economic impact is also significant, with developing countries spending approximately USD 110 billion annually on parasitic and infectious diseases resulting from and attributable to poor or inadequate personal hygiene on the part of food handlers, making this an important public health concern [13,14]. Identified risk factors include poor hygiene practices, insufficient knowledge, inadequate training, and weak enforcement of food safety regulations [7,15]. Parasitic diseases and infections spread by food handlers with poor personal hygiene remain significant public health concerns. Approximately 75% of foodborne disease outbreaks have been attributed to improper food handling, with the hands of food handlers identified as major vectors for pathogen transmission. Alarmingly, approximately 70% of foodborne illness outbreaks are linked to food service establishments, and epidemiological data suggest that mortality rates from hospital-related foodborne outbreaks are three times higher than those in other settings [16,17]

In Saudi Arabia, between 2017 and 2023, a total of 42,079 infectious foodborne illness cases were reported. The overall mean crude incidence rate was 18.0 ± 5.7 cases per 100,000 population. Of the reported cases, 12,710 were associated with identified foodborne outbreaks, while the remaining 29,369 were classified as sporadic. The most frequently detected pathogens during this period were *Entamoeba* spp., accounting for 14,796 cases, and *Salmonella* spp., with 13,130 reported cases [18,19]. Food safety oversight falls under the Saudi Food and Drug Authority (SFDA) and the Saudi Standards, Metrology and Quality Organization (SASO), which enforce food safety standards, including Good Manufacturing Practices (GMP) and Hazard Analysis and Critical Control Points (HACCP) systems. Despite these efforts, food safety implementation within healthcare facilities remains uneven, posing heightened risks to hospitalized patients who are often immunocompromised and highly vulnerable to infections [4]. Given the increased vulnerability of hospital patients due to compromised immunity, food safety in healthcare environments demands greater emphasis than is currently provided [12,13]. To address these concerns, improving food safety awareness among healthcare workers is critical. Regular training programs and adequate resources are necessary to improve knowledge and practices related to food safety within hospitals. Accordingly, this study was designed to assess the knowledge and practices related to food safety among nutrition services employees working in hospitals across the Al-Ahsa Governorate, Saudi Arabia.

## 2. Materials and Methods

### 2.1. Study Design and Participants

This descriptive cross-sectional survey aimed to assess the Knowledge and Practices of Food Safety (KPFS) among nutrition services staff in hospitals across the Al-Ahsa region. The study was conducted in five public hospitals, which are among the largest and most prominent healthcare facilities in the city. These hospitals were selected due to their high patient volume, diverse healthcare services, and significant role in providing nutrition services, making them representative sites for assessing food safety knowledge and practices within hospital environments. Participants were comprehensively selected from each department based on their voluntary consent, confirmed by signing an informed consent form. It was explicitly communicated that the collected data would be used solely for scientific research purposes. The study was conducted between November 2023 and February 2024, targeting employees in various roles within the nutrition services departments, including those responsible for supervision, food preparation, cleaning, and other food-handling tasks. Data collection was carried out using a structured electronic questionnaire specifically designed to assess participants’ awareness and practices related to food safety. The study included 302 employees, aged 18 to 60 years, working in hospitals and directly involved in food preparation, serving, and supervision of patient meals.

### 2.2. Questionnaire Design

Data for this study were collected using a structured electronic questionnaire designed to assess food safety knowledge and practices among hospital nutrition services employees. The questionnaire was primarily administered electronically, allowing participants to complete it independently through links shared via email and WhatsApp. For participants who were not fluent in Arabic or English, trained researchers conducted the questionnaire face to face to ensure accurate completion. These interviews followed the same structured format as the electronic version, maintaining consistency in data collection. This dual approach ensured broad accessibility and reliable responses across diverse linguistic backgrounds, enhancing the inclusivity and validity of the study. The survey was designed to assess food handlers’ knowledge, attitudes, and practices regarding food safety. During questionnaire development, all of the items were thoroughly reviewed to ensure their relevance to the study objectives.

The questionnaire consisted of 41 questions divided into five sections. The first section focused on demographic characteristics, including gender, age, educational level, job title, work experience, and participation in food safety training courses. The second section assessed knowledge of food safety principles, such as understanding critical temperatures, cross-contamination prevention, and hygiene practices. The third section examined personal hygiene practices, including awareness and adherence to handwashing, appropriate attire, and behaviors affecting food safety. The fourth section covered food storage and handling, emphasizing proper storage practices, the separation of raw and cooked foods, and measures to prevent cross-contamination. The final section addressed cleaning and sterilization procedures, evaluating awareness of equipment and surface sanitation, as well as the importance of maintaining a hygienic environment. Before data collection, ethical approval was obtained from the Deanship of Scientific Research (reference number KFU-REC-2023-OCT-ETHICS1585).

To ensure the accuracy and appropriateness of the data collection tool, the questionnaire was pretested with 30 nutrition services employees from one of the participating hospitals. The pretest focused on evaluating item clarity, relevance, and ease of understanding. Based on participant feedback, several adjustments were made to improve the wording and structure of specific questions. Following the pilot phase, construct validity was established through expert review by specialists in food safety, nutrition, and public health. These experts confirmed that the questionnaire items were aligned with the study’s objectives and effectively measured food safety knowledge, attitudes, and practices. Cronbach’s alpha was calculated for the knowledge and practice sections of the questionnaire to assess the internal consistency of the instrument. The overall Cronbach’s alpha value was 0.84, indicating a good level of internal reliability. These steps ensured that the final instrument was both valid and reliable for use among hospital nutrition services staff.

### 2.3. Statistical Analysis

Data were collected using Microsoft Excel and analyzed using SPSS Version 23. Descriptive statistical methods, including frequencies, percentages, means, and standard deviations, were employed to summarize the study variables. The Shapiro–Wilk test was conducted to evaluate the variable distribution. To assess the awareness of health sector workers regarding nutrition-related practices, a four-point Likert scale was utilized. Various statistical tests were conducted to examine differences in awareness levels across demographic groups. The chi-square (χ^2^) test was used to assess associations between categorical variables, while one-way ANOVA and the independent sample *t*-test were applied to compare mean awareness levels across demographic groups. Additionally, Multiple Linear Regression Analysis was conducted to evaluate the relationship between demographic factors and employees’ food safety awareness levels. A significance level of *p* < 0.05 was applied in all analyses to determine statistical significance, ensuring that the findings were robust and reliable.

## 3. Results

### 3.1. Characteristics of the Study Participants

Table 1 shows the characteristics of the study participants. A total of 302 participants were included, with 102 (33.77%) being female and 200 (66.23%) male. In terms of age distribution, the majority of participants (51.32%) were between 31 and 40 years old, followed by 24.17% in the 41–50 age group, 20.20% in the 18–30 age range, and 4.30% in the 51–60 age group. Regarding educational background, 50.66% of participants had completed secondary education, 27.81% held a bachelor’s degree, 17.22% had a diploma, and 4.30% had obtained a master’s degree. Work experience varied, with 39.40% having 1 to 5 years of experience, 32.12% with 6 to 10 years, 26.82% having more than 15 years of experience, and only 1.66% reporting no prior experience. Participants held various job titles, with the largest proportion being nutritionists (29.1%), followed by food distributors (24.5%), sanitation workers (18.9%), cooks (11.6%), food preparers (6.6%), nutrition technicians (4.3%), and other roles (5.0%). Most participants (94.37%) possessed a professional health card, while 5.63% did not. Additionally, 64.57% had attended a food safety training course, such as HACCP, whereas 35.43% had not received such training.

### 3.2. Participant’s Knowledge of Food Safety Principles

Table 2 shows the participants’ knowledge of food safety principles, including critical temperatures, prevention of cross-contamination, and hygiene practices. A majority of participants demonstrated strong agreement with fundamental food safety practices. Nearly all participants (99.67%) agreed that washing hands before starting work reduces the risk of food contamination (3.99 ± 0.17, ꭓ^2^ = 298.01, *p* = 0.001). Similarly, all participants (100%) agreed on the necessity of wearing a work uniform, hair cover, gloves, and a mask while handling food (Mean ± SD: 4.00 ± 0.00). Regarding the effectiveness of washing utensils with detergents in removing contamination, 94.37% of participants agreed (3.92 ± 0.36, ꭓ^2^ = 775.90, *p* = 0.001). Additionally, 92.38% correctly identified that perishable foods should be refrigerated at 5 °C or frozen at −18 °C (3.82 ± 0.68, ꭓ^2^ = 474.28, *p* = 0.001).

Awareness of proper hot food storage was slightly lower, with 81.13% agreeing that hot ready-to-eat food should be maintained at 65 °C (3.49 ± 1.10, ꭓ^2^ = 523.06, *p* = 0.001). Similarly, 62.25% agreed that heat kills all bacteria that cause foodborne diseases, but 16.56% remained neutral, and 7.62% disagreed (3.27 ± 1.08, ꭓ^2^ = 228.51, *p* = 0.001).

Participants’ knowledge about specific foodborne pathogens showed variability. While 59.93% agreed that Salmonella is transmitted through food, 36.75% were unsure (2.86 ± 1.43, ꭓ^2^ = 146.82, *p* = 0.001). Furthermore, 67.55% acknowledged that not all contaminated foods show visible changes in color, smell, or taste, but 16.56% did not know (3.25 ± 1.17, ꭓ^2^ = 298.23, *p* = 0.001). The concept of cross-contamination was well understood, with 98.68% agreeing that microorganisms can transfer from contaminated food via workers’ hands or kitchen utensils. Similarly, 97.02% agreed that diseases can be transmitted from an infected person through contact with food. When evaluating food storage knowledge, 94.04% agreed that undercooked meat is more susceptible to contamination than raw vegetables (Mean ± SD: 3.88 ± 0.53, ꭓ^2^ = 768.04, *p* = 0.001), and 89.07% correctly identified that raw meat should be stored on the bottom shelf of the freezer (3.79 ± 0.65, ꭓ^2^ = 661.97, *p* = 0.001).

The study also evaluated workplace hygiene, and 99.67% of the participants agreed that workers suffering from contagious skin diseases should take time off (4.00 ± 0.06, ꭓ^2^ = 298.01, *p* = 0.001), and 100% supported the evaluation of workers’ health status before hiring (4.00 ± 0.00). Finally, 92.72% of participants acknowledged that poor hygiene in meal preparation poses health risks to patients (3.87 ± 0.50, ꭓ^2^ = 738.76, *p* = 0.001).

### 3.3. Assessing Employees’ Knowledge and Commitment to Personal Hygiene Standards

According to the Likert scale analysis in Table 3, the employees demonstrated a high level of agreement regarding personal hygiene practices critical to food safety. For the statement “Wearing gloves is important to reduce the risk of food contamination,” 99.67% of employees agreed, with an average score of 4.00 ± 0.06, and the chi-square test indicated a statistically significant result (ꭓ^2^ = 298.01, *p* = 0.001). Similarly, for “Wearing a mask is important to reduce the risk of food contamination,” 99.67% of participants agreed, achieving the same average score of 4.00 ± 0.06 and a significant chi-square value (ꭓ^2^ = 298.01, *p* = 0.001). Regarding the importance of “Wearing a head covering and work clothes to reduce the risk of food contamination,” full consensus was observed, with 100% agreement and an average score of 4.00 ± 0.00. In assessing the statement “Food handlers should not touch food if they have wounds on their hands without gloves,” 98.68% of employees agreed, with an average score of 3.98 ± 0.2; the chi-square value was significant (ꭓ^2^ = 580.25, *p* = 0.001). Lastly, when evaluating the belief that “Long and colored nails contaminate food and cause foodborne diseases,” 98.68% of employees agreed, achieving an average score of 3.97 ± 0.26, with a significant chi-square value (ꭓ^2^ = 580.23, *p* = 0.001). These findings confirm that the workforce exhibits a very high level of understanding and commitment to key personal hygiene practices essential for ensuring food safety.

### 3.4. Assessing Employees’ Knowledge of Food Storage and Cross-Contamination Prevention

As shown in Table 4, the Likert scale analysis showed a high level of staff knowledge regarding proper food storage practices and methods to prevent cross-contamination. For the statement “Properly sterilize knives and cutting boards to prevent cross-contamination,” 99.34% of staff agreed, with an average score of 3.99 ± 0.08, and the chi-square test confirmed statistical significance (ꭓ^2^ = 294.05, *p* = 0.001). Regarding the statement that “Dish towels are a potential source of food contamination,” 97.02% of staff agreed, with an average score of 3.95 ± 0.30 and a highly significant chi-square result (ꭓ^2^ = 835.61, *p* = 0.001). Similarly, for “Checking the temperature of refrigerators periodically to reduce the risk of food contamination,” 97.68% of employees agreed, achieving an average score of 3.95 ± 0.36 and a significant chi-square value (ꭓ^2^ = 562.73, *p* = 0.001). In response to “Storing raw and cooked foods separately to reduce the risk of food contamination,” 97.35% agreed, with an average score of 3.94 ± 0.39 and confirmed statistical significance (ꭓ^2^ = 556.97, *p* = 0.001).

However, for the statement “The ideal way to thaw chicken quickly is to place it in an airtight bag and then in a bowl of cold water”, a lower agreement rate was observed, with 68.87% agreeing, and a relatively lower average score of 3.25 ± 1.19. This item showed a neutral overall trend despite being statistically significant (ꭓ^2^ = 320.67, *p* = 0.001). Finally, regarding “Foods that have been defrosted should not be re-frozen”, 95.03% of employees agreed, with an average score of 3.88 ± 0.57 and a highly significant chi-square value (ꭓ^2^ = 790.47, *p* = 0.001). Overall, the general trend across all items showed strong agreement among staff (mean score 3.83 ± 0.35), indicating a good level of knowledge about food storage and practices to prevent cross-contamination.

### 3.5. Impact and Correlation of Demographic Factors on Food Safety Awareness and Knowledge Among Nutrition Services Workers

**Figure 1:**
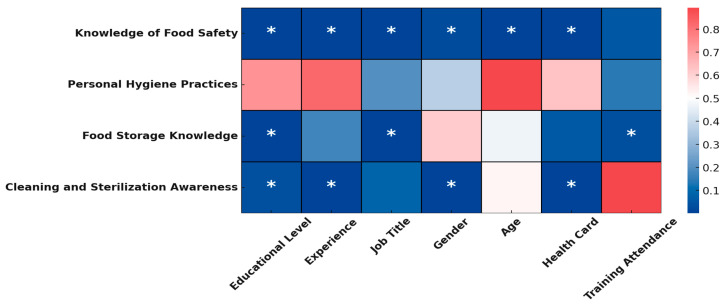
Heatmap showing the relationship between demographic characteristics and various aspects of food safety knowledge and practices. Darker blue shades represent stronger associations, while lighter and red shades indicate non-significant associations. Asterisks (*) denote statistically significant relationships (*p* < 0.05) between demographic factors.

A heatmap (Figure 1) was constructed to illustrate the relationship between various demographic characteristics and food safety knowledge and practices. The findings indicate that all demographic factors had a statistically significant (*p* ≤ 0.05) effect on employees’ knowledge of food safety principles, critical temperatures, cross-contamination prevention, and hygiene practices. Regression analysis revealed that age, job title, and possession of a health card positively influenced knowledge, whereas gender, experience, and educational level did not show a significant impact. Regarding personal hygiene practices, none of the demographic variables significantly affected employees’ understanding or commitment, indicating a uniformly high level of awareness among all workers. In terms of food storage knowledge, educational level, job title and attendance at food safety training courses were statistically significant (*p* ≤ 0.05) related positives associated with better knowledge, while other demographic factors had no significant influence. Awareness of cleaning and sterilization procedures was significantly associated with educational level, experiences, gender and possession of a health card, emphasizing the importance of certification and specialized training related to food hygiene. A comprehensive analysis of the data from different study areas showed that demographic factors like age, job title, and health card possession enhance food safety knowledge. The consistently high commitment to personal hygiene practices across demographics highlights strong baseline training in these areas.

## 4. Discussion

This study assessed the knowledge and practices related to food safety among nutrition services employees in hospitals across Al-Ahsa Governorate, with a sample size of 302 employees. The results show a generally high level of knowledge regarding food safety principles, critical temperatures, cross-contamination prevention, and hygiene practices, reflected by an average score of 3.74. This indicates that employees are well aware of key food safety concepts essential for maintaining public health standards in hospital nutrition services. These findings are consistent with previous studies [20,21]. Recently, a study by Abdelwahed et al. reported that 98% of food handlers had a positive attitude towards food safety and temperature control [21]. Similarly, a study by Alsultan found that food handlers in Riyadh hospitals exhibited good knowledge and practices [22], while a study by Hamed and Mohammed showed that a significant proportion of participants demonstrated good food safety knowledge and positive attitudes [23]. These similarities suggest that nutrition services employees, both locally and in other regions, tend to have a strong understanding of fundamental food safety principles. However, the results diverge from findings reported in other studies. Moreover, a previous study by Abdelwahed highlighted that over a quarter of food handlers demonstrated poor knowledge [21], while a study by Teffo and Tabit in South Africa revealed a lack of awareness regarding correct food-handling temperatures among hospital workers [24]. Additionally, another study by Al-Mohaithef et al. showed poor understanding of critical food safety temperatures among restaurant supervisors in Dammam [25]. These differences could be attributed to variations in training quality, institutional policies, or enforcement of food safety standards between regions and facilities. Significant differences in knowledge were observed based on educational level and job title. Employees with secondary education reported higher agreement with food safety principles, and nutritionists and food distributors demonstrated significantly greater knowledge compared to those with other job titles (*p* = 0.001). This finding aligns with a study conducted by Teffo and Tabit, which suggested that while education and experience are important, the nature of job roles also strongly influences food safety practices [24]. It also aligns with a systematic review, which found that, while knowledge was moderate among Bangladeshi hospital workers, attitudes and practices were notably strong [26]. In contrast, other studies, such as those by El-Gamal et al. and Nyawo et al., found low levels of food safety knowledge among food handlers, often linked to insufficient education and lack of mandatory training [26,27]. These findings highlight the critical need for continuous training programs, even when baseline knowledge is relatively high.

Notably, 64.57% of employees in this study had attended food safety training courses, reflecting a strong commitment to professional development. This is in sharp contrast to the study conducted by Teffo and Tabit, where only 27.6% of hospital food handlers reported receiving food safety training [24]. This difference underscores the positive impact that mandatory, structured training can have on food safety awareness. Employee commitment to personal hygiene practices was exceptionally high, with an average score of 3.99. Practices such as handwashing, wearing appropriate clothing, and avoiding unsafe behaviors were widely adhered to. These results are supported by the study conducted in Al-Suwaira city in Iraq in 2023 and the study conducted in Kuwait by Moghnia et al. in 2021, which also reported high compliance rates with personal hygiene standards among food handlers [28,29]. However, their findings contrast with those of Hafez et al. (2022), which showed gaps in personal hygiene knowledge and practices among hospital food handlers in Egypt [30]. Regarding food storage and cross-contamination prevention, employees demonstrated strong knowledge, with an average score of 3.83. This is consistent with findings from previous studies that reported high levels of understanding and knowledge of correct practices among food service workers in hospital settings [25,31,32]. Nevertheless, some variation exists, as observed in the study that indicated weaknesses in food storage knowledge among certain groups [28].

Finally, the study found very high awareness regarding cleaning and sterilization procedures, with an average score of 3.98. This reflects a critical understanding of the importance of maintaining a clean and healthy environment, which is in line with the findings of a study that showed satisfactory levels of food safety knowledge among street food handlers in Ghana [32]. However, contrasting findings from studies reported that poor hygiene practices remain an issue in other contexts, pointing to the need for continual education and supervision [28,33,34]. Overall, the results of this study demonstrate that nutrition services employees in Al-Ahsa hospitals have a strong foundation of food safety knowledge and hygiene practices. However, ongoing training and reinforcement remain essential to ensure that these high standards are consistently maintained and improved.

## 5. Conclusions

The results of this study indicate that nutrition services employees in hospitals across the Al-Ahsa Governorate possess a high level of knowledge regarding food safety principles, critical temperatures, the prevention of cross-contamination, and proper hygiene practices. The employees also showed strong commitment to personal hygiene standards, food storage protocols, and equipment sterilization procedures. These findings highlight the effectiveness of current food safety education and training programs implemented in hospital settings, underscoring the Ministry of Health’s clearly defined and strictly enforced roles in overseeing, regulating, and ensuring adherence to food safety standards in Saudi Arabia. However, variations in knowledge based on job title highlight the need for targeted training to further enhance food safety practices. The overall high level of awareness and compliance underscores the importance of maintaining structured, continuous education to preserve and improve these standards. Based on the study’s findings, hospitals are advised to enhance and routinely update food safety training programs, ensuring that all nutrition services employees maintain high standards of knowledge and practice. Special attention should be given to areas where knowledge gaps were identified, such as proper thawing methods, to further strengthen employee competence. Continuous monitoring and periodic assessments should be established to evaluate employees’ adherence to food safety protocols and identify areas requiring additional support. Requiring employees to obtain and renew professional health cards would help sustain high food safety awareness, as the possession of such certification was positively associated with better practices. Furthermore, training programs should be customized according to employees’ job roles, ensuring that the educational content is practical and relevant to their specific duties. Finally, fostering a strong culture of food safety within hospitals, through leadership support and peer engagement, would reinforce individual responsibility and commitment to maintaining high hygiene and food safety standards.

## 6. Strengths and Limitations

This study has several strengths. It was conducted on a relatively large and diverse sample of 302 nutrition services employees across multiple hospitals in Al-Ahsa Governorate, providing a comprehensive understanding of food safety knowledge and practices within a healthcare setting. The use of a structured questionnaire allowed for standardized data collection and enabled the identification of specific areas of strength and weakness among employees. Furthermore, comparing the study findings with previous national and international research added depth to the analysis and positioned the results within a broader context.

This study also has some limitations. It relied on self-reported data, which may introduce bias, as participants might provide socially desirable responses rather than fully accurate ones. Additionally, the cross-sectional design limits the ability to establish causal relationships between demographic factors and food safety knowledge and practices. The study sample was also geographically limited to hospitals in one region, which may affect the generalizability of the findings to other regions or healthcare settings with different training standards or cultural factors. Furthermore, all participating hospitals were large public institutions, and no comparison was made with smaller or private hospitals. This limits the ability to assess how institutional type, or organizational structure may influence food safety practices and awareness. Future studies should aim to include a broader range of hospital types to explore potential contextual or structural differences.

Finally, while the study explored general knowledge and practices, it did not directly observe behaviors in real workplace settings, which could provide additional validation of the self-reported data. Future research should consider integrating such objective measures to validate self-reported knowledge and better understand the link between knowledge and behavior in food safety management.

## Figures and Tables

**Table 1:** Characteristics of the study participants.

Variable		Number	Percentage (%)
Sex	Female	102	33.77
	Male	200	66.23
Age (in years)	18–30	61	20.20
	31–40	155	51.32
	41–50	73	24.17
	51–60	13	4.30
Educational level	Secondary	153	50.66
	Diploma	52	17.22
	Bachelor’s	84	27.81
	Master’s	13	4.30
Experience	No experience	5	1.66
	From 1 to 5 years	119	39.40
	6–10 years	97	32.12
	More than 15 years	81	26.82
Job title	Sanitation worker	57	18.9
	Food distributor	74	24.5
	Food preparer	20	6.6
	Cook	35	11.6
	Nutrition technician	13	4.3
	Nutritionist	88	29.1
	Other	15	5.0
Do you have a professional card (health card)?	No	17	5.63
	Yes	285	94.37
Have you attended any training course in food safety? like HACCP or other	No	107	35.43
	Yes	195	64.57

HACCP, Hazard Analysis and Critical Control Points.

**Table 2:** Likert scale analysis for assessing employees’ knowledge of food safety principles, critical temperatures, prevention of cross-contamination, and proper hygiene practices.

Variables		Number	Percentage (%)	Mean ± SD	Direction	ꭓ^2^	Sig.
1-It is necessary to wash hands before starting work because it reduces the risk of food contamination.	I don’t know	1	0.33	3.99 ± 0.17	I agree	298.01	0.001
	I do not agree	0	0				
	Neutral	0	0				
	I agree	301	99.67				
2-It is necessary to wear work uniform, hair cover, gloves and mask while handling food.	I don’t know	0	0	4.00 ± 0.00	I agree	NP	NP
	I do not agree	0	0				
	Neutral	0	0				
	I agree	302	100				
3-Utensils washed with special detergents will be free from contamination.	I don’t know	2	0.66	3.92 ± 0.36	I agree	775.90	0.001
	I do not agree	3	0.99				
	Neutral	12	3.97				
	I agree	285	94.37				
4-The correct temperatures for storing perishable foods are refrigerated at 5 °C or frozen at −18 °C.	I don’t know	16	5.30	3.82 ± 0.68	I agree	474.28	0.001
	I do not agree	0	0				
	Neutral	7	2.32				
	I agree	279	92.38				
5-Hot food ready to eat must be kept at a temperature of 65 °C.	I don’t know	47	15.56	3.49 ± 1.10	I agree	523.06	0.001
	I do not agree	3	0.99				
	Neutral	7	2.32				
	I agree	245	81.13				
6-Heat kills all types of bacteria that cause foodborne diseases.	I don’t know	41	13.58	3.27 ± 1.08	I agree	228.51	0.001
	I do not agree	23	7.62				
	Neutral	50	16.56				
	I agree	188	62.25				
7-Salmonella bacteria are bacteria that are transmitted through some foods.	I don’t know	111	36.75	2.86 ± 1.43	Neutral	146.82	0.001
	I do not agree	0	0				
	Neutral	10	3.31				
	I agree	181	59.93				
8-Not all contaminated foods contain some change, such as a change in color, smell, or taste.	I don’t know	50	16.56	3.25 ± 1.17	Neutral	298.23	0.001
	I do not agree	29	9.60				
	Neutral	19	6.29				
	I agree	204	67.55				
9-Microorganisms are transferred from contaminated food by workers’ hands or kitchen utensils to other food.	I don’t know	0	0	3.98 ± 0.21	I agree	580.25	0.001
	I do not agree	3	0.99				
	Neutral	1	0.33				
	I agree	298	98.68				
10-The disease can be transmitted from an infected person through contact with food.	I don’t know	1	0.33	3.96 ± 0.25	I agree	835.74	0.001
	I do not agree	1	0.33				
	Neutral	7	2.32				
	I agree	293	97.02				
11-Undercooked meat is more susceptible to contamination than raw vegetables.	I don’t know	8	2.65	3.88 ± 0.53	I agree	768.04	0.001
	I do not agree	2	0.66				
	Neutral	8	2.65				
	I agree	284	94.04				
12-The appropriate place to store raw meat in the freezer is the bottom shelf.	I don’t know	7	2.32	3.79 ± 0.65	I agree	661.97	0.001
	I do not agree	17	5.63				
	Neutral	9	2.98				
	I agree	269	89.07				
13-When suffering from a contagious skin disease, it is necessary to take time off from work.	I don’t know	0	0	4.00 ± 0.06	I agree	298.01	0.001
	I do not agree	0	0				
	Neutral	1	0.33				
	I agree	301	99.67				
14-The health status of workers must be evaluated before hiring procedures.	I don’t know	0	0	4.00 ± 0.00	I agree	NP	NP
	I do not agree	0	0				
	Neutral	0	0				
	I agree	302	100				
15-The meals that are served pose health risks due to poor hygiene for patients.	I don’t know	4	1.32	3.87 ± 0.5	I agree	738.76	0.001
	I do not agree	9	2.98				
	Neutral	9	2.98				
	I agree	280	92.72				

Sig: Significant level of *p* < 0.05.

**Table 3:** Likert analysis for assessing employees’ understanding of and adherence to personal hygiene practices.

Variables		Number	Percentage (%)	Average	Direction	ꭓ^2^	Sig.
1-Wearing gloves is important to reduce the risk of food contamination	I don’t know	0	0	4.00 ± 0.06	I agree	298.01	0.001
	I do not agree	0	0				
	Neutral	1	0.33				
	I agree	301	99.67				
2-Wearing a mask is important to reduce the risk of food contamination	I don’t know	0	0	4.00 ± 0.06	I agree	298.01	0.001
	I do not agree	0	0				
	Neutral	1	0.33				
	I agree	301	99.67				
3-Wearing a head covering and work clothes is an important practice to reduce the risk of food contamination	I don’t know	0	0	4.00 ± 0.00	I agree		NP
	I do not agree	0	0				
	Neutral	0	0				
	I agree	302	100				
4-Food handlers should not touch food if they have wounds on their hands without gloves	I don’t know	1	0.33	3.98 ± 0.2	I agree	580.25	0.001
	I do not agree	0	0				
	Neutral	3	0.99				
	I agree	298	98.68				
5-Long and colored nails contaminate food, causing foodborne diseases	I don’t know	2	0.66	3.97 ± 0.26	I agree	580.23	0.001
	I do not agree	0	0				
	Neutral	2	0.66				
	I agree	298	98.68				

Sig: Significant level of *p* < 0.05.

**Table 4:** Likert scale analysis for assessing staff knowledge of proper food storage practices and prevention of cross-contamination during handling.

Variables		Number	Percentage (%)	Average	Direction	ꭓ^2^	Sig.
1-Properly sterilize knives and cutting boards to prevent cross-contamination	I don’t know	0	0	3.99 ± 0.08	I agree	294.05	0.001
	I do not agree	0	0				
	Neutral	2	0.66				
	I agree	300	99.34				
2-Dish towels are a potential source of food contamination	I don’t know	2	0.66	3.95 ± 0.3	I agree	835.61	0.001
	I do not agree	1	0.33				
	Neutral	6	1.99				
	I agree	293	97.02				
3-Check the temperature of refrigerators periodically to reduce the risk of food contamination	I don’t know	4	1.32	3.95 ± 0.36	I agree	562.73	0.001
	I do not agree	0	0				
	Neutral	3	0.99				
	I agree	295	97.68				
4-Store raw and cooked foods separately to reduce the risk of food contamination	I don’t know	5	1.66	3.94 ± 0.39	I agree	556.97	0.001
	I do not agree	0	0				
	Neutral	3	0.99				
	I agree	294	97.35				
5-The ideal way to thaw chicken quickly is to place it in an airtight bag and then in a bowl of cold water	I don’t know	52	17.22	3.25 ± 1.19	neutral	320.67	0.001
	I do not agree	30	9.93				
	Neutral	12	3.97				
	I agree	208	68.87				
6-Defrosted foods should not be refrozen	I don’t know	10	3.31	3.88 ± 0.57	I agree	790.47	0.001
	I do not agree	2	0.66				
	Neutral	3	0.99				
	I agree	287	95.03				
	Mean and deviation			0.35 ± 3.83	General trend	I agree	

Sig: Significant level of *p* < 0.05.

## Data Availability

The data presented in this study are available on request from the corresponding author.

## Abbreviations

The following abbreviations are used in this manuscript:

KPFS	Knowledge and Practices related to Food Safety.
SFDA	Saudi Food and Drug Authority.
SASO	Saudi Standards, Metrology and Quality Organization.
GMP	Good Manufacturing Practices.
HACCP	Hazard Analysis and Critical Control Points.
